# Scaling and Accommodation of Jaw Adductor Muscles in Canidae

**DOI:** 10.1002/ar.23355

**Published:** 2016-05-11

**Authors:** Fay Penrose, Graham J. Kemp, Nathan Jeffery

**Affiliations:** ^1^ Institute of Ageing and Chronic DiseaseDepartment of Musculoskeletal Biology and the MRCArthritis Research UK Centre for Integrated Research into Musculoskeletal Ageing (CIMA)University of LiverpoolLiverpoolUnited Kingdom; ^2^ School of Veterinary ScienceDepartment of Veterinary Preclinical ScienceUniversity of LiverpoolLiverpoolUnited Kingdom; ^3^ Human Anatomy Resource CentreUniversity of LiverpoolLiverpoolUnited Kingdom

**Keywords:** canid, hypercarnivorous, jaw adductor, muscle, scaling, accommodation

## Abstract

The masticatory apparatus amongst closely related carnivoran species raises intriguing questions about the interplay between allometry, function, and phylogeny in defining interspecific variations of cranial morphology. Here we describe the gross structure of the jaw adductor muscles of several species of canid, and then examine how the muscles are scaled across the range of body sizes, phylogenies, and trophic groups. We also consider how the muscles are accommodated on the skull, and how this is influenced by differences of endocranial size. Data were collected for a suite of morphological metrics, including body mass, endocranial volume, and muscle masses and we used geometric morphometric shape analysis to reveal associated form changes. We find that all jaw adductor muscles scale isometrically against body mass, regardless of phylogeny or trophic group, but that endocranial volume scales with negative allometry against body mass. These findings suggest that head shape is partly influenced by the need to house isometrically scaling muscles on a neurocranium scaling with negative allometry. Principal component analysis suggests that skull shape changes, such as the relatively wide zygomatic arches and large sagittal crests seen in species with higher body masses, allow the skull to accommodate a relative enlargement of the jaw adductors compared with the endocranium. Anat Rec, 299:951–966, 2016. © 2016 The Authors The Anatomical Record: Advances in Integrative Anatomy and Evolutionary Biology Published by Wiley Periodicals, Inc.

Interspecific differences of Carnivoran skull shape are dependent on numerous factors, most notably phylogeny, dietary function and allometry with the relative importance of each depending on the group of species under investigation. Here, we attempt to resolve the relative importance of allometry and diet in determining cranial morphology among one particularly widespread and varied carnivoran family, the canids. We aim to account for phylogeny and determine how labile the musculoskeletal morphology of the wild canid head is by combining advances in imaging with conventional dissection and more advanced computational methods such as geometric morphometrics. In contrast to many previous studies (Christiansen and Adolfssen 2005; Wroe and Milne 2007; Figueirido et al., 2011; Damasceno et al., 2013) we directly quantify the masticatory muscles as well as the bony morphology.

Radinsky (1981) was amongst the first to document that carnivoran skull shape is linked to negative allometric scaling of the brain among related species but did not consider, in detail, questions concerning the potential knock‐on effects for the masticatory apparatus. In particular, are the areas for muscle origin on the skull compromised with the relative reduction of brain size and of the surrounding neurocranium, and does this influence the size of the muscle mass that can be accommodated? In addition, is this further compounded by the positive allometric scaling of the masticatory muscles needed to maintain the same level of biomechanical function? Emerson and Bramble (1993) state that large species can exert relatively less muscle force than small species, and are required to move relatively and absolutely heavier jaws. This implies that with increases of body size, species either lose function or must have relatively larger muscles that in turn require a commensurate increase in the bony areas for their attachments. Numerous studies have also linked skull form with dietary function (Sacco and Van Valkenburgh, 2004; Meachen‐Samuels and Van Valkenburgh 2009; Tseng and Wang 2010; Sicuro and Oliveira 2011; Tseng and Anton, 2011). Slater and Van Valkenburgh (2008, 2009) have shown that big cats have lengthened their jaw to facilitate a relatively wider gape than small cats. This suggests that big cats are not simply “scaled up” small cats, but make different functional demands of their jaws. This morphological difference coincides with a difference in their diet and hunting strategies; whereas small cats take prey smaller than themselves, big cats require a relatively wider gape to subdue prey which may be larger than them (Slater and Van Valkenburgh, 2009).

Here we look collectively at the scaling of brain size, masticatory muscle size and trophic niche as determinants of canid skull morphology. Canids were selected for the present study because they are diverse in body mass, geographical location, and dietary group specialization and their phylogeny is relatively well documented (Gittleman, 1985; Wayne et al., 1989; MacDonald and Sillero‐Zubiri, 2004; Sillero‐Zubiri et al., 2004; Finarelli, 2007; Macdonald, 2009; Wang and Tedford, 2010). All 36 species (Nowak, 2005) of extant canids, the canidae, belong to the subfamily caninae and are thought to have evolved from a common ancestor that originated in North America around 8–12 million years ago (Wang and Tedford, 2010). Modern species are arranged in four main phylogenetic clades, the fox‐like vulpes clade, the wolf‐like canis clade, the South American clade and the grey fox‐like Urocyon clade (Lindblad‐Toh et al., 2005). Both convergent and divergent patterns of morphological adaptation are found within and amongst these clades. For instance, the South American foxes, although phenotypically very similar to the fox‐like vulpes are more closely genetically related to the wolf‐like canids (Wayne et al., 1997; Perini et al., 2010; Nyakatura and Bininda‐Emonds, 2012). Conversely, morphologies amongst closely related species such as the South American *Speothos venaticus* and *Chrysocyon brachyurus* are very distinct and highlight a great potential for phenotypic plasticity. Three trophic groups exist which allow us to correlate head morphology with hunting behavior and functional dietary requirements; these are the small prey specialists, the generalists and the large prey specialists. These dietary specialisms are not dictated by phylogenetic clade: the fox‐like group consists both of generalists and small prey specialists, the South American group of generalists and small and large prey specialists and the wolf‐like group of generalists and large prey specialists. Both of the urocyon clade members are generalists (Slater et al., 2009).

## AIMS OF THE STUDY

Scaling of masticatory muscle masses, as opposed to bony proxies, is not widely described in many species of mammal but previous studies have established that there is no common rule regarding the relative size of the jaw adductors within clades. Primates demonstrate isometric scaling regardless of diet or phylogeny (Cachel, 1984; Perry and Wall, 2008). Herrel describes the mass of the temporalis muscles of a wide range of bats, including frugivorous, insectivorous and sanguivorous species, scaling with negative allometry (Herrel et al., 2008). Macropodoideal marsupials show a range of scaling patterns in all jaw adductors, according to dietary preference (Warburton, 2009). Similarly, the relative masseter muscle mass in ruminants has been shown to differ amongst species with different feeding categories independent of body mass or phylogeny (Clauss et al., 2008). Within the carnivoran order Hartstone–Rose established that the masticatory muscle masses scale with isometry that tends towards positive allometry (Hartstone‐Rose et al., 2012). Here we aim to describe the jaw adductor muscles of several species of canid and establish whether they scale isometrically against body mass, or more closely follow other patterns that reflect dietary function or phylogeny. Specifically, we will consider how temporalis, masseter, and the pterygoids contribute to the entire jaw adductor mass, their gross architecture, their mass compared to body mass and to endocranial volume, and their specific and relative areas of attachment to the skull. We also evaluate the hypothesis that species with a high bite force and large body mass, such as the hypercarnivores (Wroe et al., 2005; Christiansen and Wroe, 2007), have absolutely and relatively larger muscles, and we speculate that the gross morphology of the masticatory musculature of hypercarnivorous canid species differs from those of generalists and small prey specialists and deviates significantly from simple predictive patterns of size scaling. As the jaw adductor muscles arise solely from the cranium and cover much of its external surface, we also consider how they are accommodated on the skull and, through shape analysis, explore whether the diversity of head shape among canids is influenced by constraints and concomitant compensatory adjustments for housing the masticatory muscles. Previous studies have been able to categorise canids according to diet based on overall skull shape (Radinsky, 1981; Van Valkenburgh, 2007) or upper jaw morphology (Slater et al., 2009) with the hypercarnivorous species tending toward a broad stocky skull and shortened snout, and the small prey specialists being more gracile with a long rostrum and narrow jaws. Here we regard the bony skeleton of the head to be made of three modules—the cranial part, the rostral part and the mandible, and consider if all of the modules aid in determining diet or if some are instead allied with muscle accommodation.

## MATERIALS AND METHODS

### Specimens

**Figure 1:**
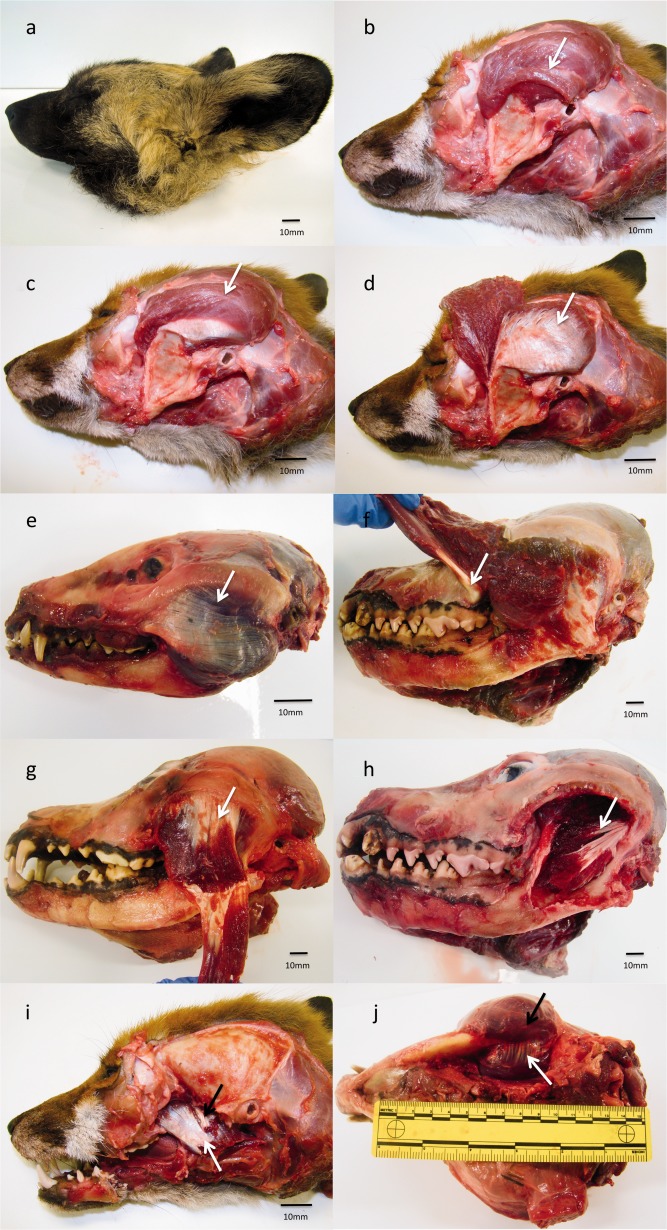
(a) *Lycaon pictus*, (**b**) *Suprazygomatic temporalis* (white arrow), *Vulpes vulpes*, (**c**) Superficial temporalis (white arrow), *Vulpes vulpes*, (**d**) Deep temporalis (white arrow), *Vulpes vulpes*. (**e**) Superficial masseter (white arrow) *Nyctereutes procyonoides*, (**f**) Tendon of origin of superficial masseter (white arrow), *Lycaon pictus*, (**g**) Deep masseter (white arrow), *Canis lupus*, (**h**) Zygomaticomandibularis (white arrow), *Lycaon pictus*. (**i**) Medial pterygoid (white arrow) and lateral pterygoid (black arrow), *Vulpes vulpes*, (**j**) Superficial masseter (black arrow), and pterygoids (white arrow), *Canis lupus*.

Specimens from 8 of the 13 genera that make up the canidae family were obtained from either euthanased zoo stock or vermin control (Table 1). For this study we follow Wozencraft and define the arctic fox as a member of the *Vulpes* genus (Wozencraft, 1993). There were 19 individuals from 12 species with representatives from the three major clades and the three trophic groups. The data set is not inclusive of all canid species, however, it covers a broad range of head shapes, body sizes and phylogenetic groups, and it includes all four of the hypercarnivorous species (Van Valkenburgh, 2007). Although numbers of specimens are low for all species in this study, diversity of scale covers two orders of magnitude in the canidae and interspecific differences are greater than intraspecific ones. For the purposes of this study species were identified as being from one of the three trophic groups as described by Slater et al. (2009) (Table 1). All specimens were adults and exact ages as recorded by donor organizations were recorded in six specimens, and maturity established for the others with reference to dental wear. In some cases only the heads were available and so mean body masses as reported in the literature were used for all calculations (Table 1). Sex was recorded in all individuals. Some degree of sexual dimorphism has been documented in many canid species, but the literature concurs that it is modest and that overall body size is the greatest differential factor. Males often have a slightly greater body mass and larger overall proportions than females, however there appears to be a significant amount of overlap in body mass data between the largest females and smallest males (MacDonald and Sillero‐Zubiri 2004; Sillero‐Zubiri et al., 2004; Macdonald 2009; Wang and Tedford 2010). Several species have also been shown to exhibit some sexual dimorphism relating to dentition, although canids, along with hyaenids, are noted to be the least dimorphic in this respect of all of the carnivores. Where species have been shown to exhibit dental dimorphism, males typically have 8–15% longer canines but this is thought to relate to behavioral displays and is not correlated to body mass or skull length (Gittleman and Van Valkenburgh, 1997; Kim et al., 2012). Specimens were either chilled fresh or frozen and then defrosted, but no fixative agent was used on any specimen. All specimens were dissected at near occlusal bite, that is, with minimal gape (Fig. 1a).

**Table 1:** Details of specimens, mean muscle masses, muscle attachment surface areas, endocranial volumes, and endocranial surface areas

	*n*/sex	Phylogenetic group	Dietary specialism	Mean body mass (g) from literature^a^, ^b^	Temporalis mass (g)	Masseter mass (g)	Pterygoid mass (g)	Total jaw adductor mass (g)	Temporalis surface area (mm^2^)	Masseter surface area (mm^2^)	Pterygoid surface area (mm^2^)	Total jaw adductor surface area (mm^2^)	Endocranial volume (mm^3^)	Endocranial volume surface area (mm^2^)
*Alopex lagopus*	1M	Fox‐like	Small prey specialist	5,200	43.8	14.9	4.47	63.1	2,298	486	329	3,113	45,290	9,360
*Canis lupus*	1F,2M	Wolf‐like	Hypercarnivore	36,500	179.5	84.5	25.7	289.7	5,593	2,069	1,397	9,059	143,367	18,575
*Canis mesomelas*	1M	Wolf‐like	Small prey specialist	9,700	46.6	20.2	6.7	73.5	2,822	682	479	3,983	67,830	11,042
*Chrysocyon brachyurus*	1F	South American	Small prey specialist	25,000	106.1	61.5	13.2	180.8	5,428	1,443	937	7,808	111,800	14,988
*Cuon alpinus*	1F	Wolf‐like	Hypercarnivore	13,500	81.6	40.6	10.4	132.6	4,383	1,197	776	6,356	108,200	16,365
*Lycaon pictus*	1F,2M	Wolf‐like	Hypercarnivore	26,500	141.7	84.4	19.4	245.5	5,853	1,611	1,311	8,775	149,500	19,877
*Nyctereutes procyonoides*	1M	Fox‐like	Generalist	6,500	19.9	10.6	3.2	33.7	2,237	632	276	3,145	30,120	7,905
*Otocyon megalotis*	1M	Fox‐like	Generalist	4,200	13.5	6.6	2.3	22.4	1,140	406	248	1,794	30,490	7,159
*Speothos venaticus*	1F	South American	Hypercarnivore	6,500	42.7	24.6	5.1	72.4	2,864	694	438	3,996	51,720	9,341
*Vulpes corsac*	3M, 1 unknown	Fox‐like	Small prey specialist	2,850	14.7	6.4	2.3	23.4	1,520	381	275	2,176	31,910	6,677
*Vulpes vulpes*	1M	Fox‐like	Small prey specialist	8,500	48	19.3	5.7	73	2,739	725	498	3,962	52,430	10,060
*Vulpes zerda*	1F	Fox‐like	Generalist	1,150	5.6	2.4	0.9	8.9	927	203	117	1,247	19,560	5,100

a Nowak, R.M 2005 (Nowak, 2005).

b Macdonald and Sillero‐Zubiri 2004 (MacDonald and Sillero‐Zubiri, 2004).

### Dissection

One side of the head, either left or right, was dissected for each specimen and then photographed using a digital camera (Sony DSC‐H200) that was positioned perpendicular to the sagittal, axial and coronal planes of each specimen. No individual was judged to have a preferential working side judging from dental wear. For the present study we limit the definition of the masticatory apparatus to the jaw adductor muscles (temporalis, masseter, and pterygoids) and associated skeletal components, although in reality other structures such as the tongue and salivary glands also contribute towards food prehension and mastication. The constituent bellies of the jaw adductor muscles were photographed *in situ* alongside a graduated rule and removed layer by layer to document architectural detail. After removal each layer was wrapped in damp cloth along with the rest of the muscle division and stored in plastic wrap to prevent dehydration. All muscle divisions were subsequently weighed using digital scales (Redwag WPS600/C/2) to determine mass. Each muscle sample was weighed three times and the average recorded. Muscle classification and nomenclature of subdivisions within each muscle varies between authors (Druzinsky et al., 2011). For this study we broadly follow the plan of Turnbull (1970) who identifies the temporalis as subdivided into suprazygomatic, superficial, and deep divisions and the masseteric muscle mass subdivided into superficial, deep, and zygomaticomandibularis divisions. Some authors define zygomaticomandibularis as the deepest division of the masseter complex (Evans and De Lahunta, 2012). In accordance with other authors (Davis, 1955; Hartstone‐Rose et al., 2012) we recognize it as subdivision of the masseter as it also arises from the zygomatic arch and inserts onto the lateral vertical ramus of the mandible. The lateral pterygoid is very small in carnivores (Turnbull, 1970; Herring, 2007) and in this study we have included it along with the much larger medial pterygoid to be considered as one muscle mass, the pterygoids. In mammals capable of rostro‐caudal and lateral movement of the jaw the lateral pterygoid is able to protract the jaw and aid in lateral translations. However, in carnivore species rostro‐caudal movement of the mandible is precluded by the cylindrical form of the mandibular fossa of the temporal bone and the well‐developed retroglenoid process. Lateral movement is also greatly limited. As a result the only movement at the temporomandibular joint in carnivores is rotation around the mandibular condyle and, consequently, the lateral pterygoid contributes to jaw closing (Getty, 1975; Ström et al., 1988; Dyce et al., 1996; Herring, 2007; König and Liebich, 2009; Evans and De Lahunta, 2012).

### Imaging

To capture the internal and external architecture of the skull in three‐dimensional detail, heads were scanned at the University of Liverpool using computer tomography (CT) either at the Small Animal Teaching Hospital using a Siemens Somatom Volume Zoom (Siemens AG, Munich) or a Toshiba Prime Aquilion (Toshiba Medical Systems, Europe), or at the Philip Leverhulme Equine Hospital using a GE Lightspeed Plus (GE Medical Systems, Milwaukee). Pixel resolution ranged from 0.136 to 0.417 mm and slice thickness from 0.3 to 1.2 mm. Current and voltages used were 120 kV and 200 mA. Preprocessing of CT data was done with ImageJ v1.45s (Schneider et al., 2012).

### Landmarking

Scans for each specimen were reconstructed in virtual 3D by label mapping in Avizo 8.1(FEI Systems, OR). Reconstructions and oblique slices were used to locate and place a series of 71 anatomical landmarks, representing the whole skull and mandible. Three subsets of landmarks were chosen to represent the three components, or modules (Klingenberg, 2009), of the bony skeleton—the neurocranium that is most closely associated with the hypothesized constraint of muscle and brain size scaling, the rostral component that is most closely associated with the nasal cavity and upper dental arcade and the mandibular component associated with housing the lower dental arcade (Appendix 1). Subsets were then used as variables in further tests.

### Volume and Area Measurements

**Figure 2:**
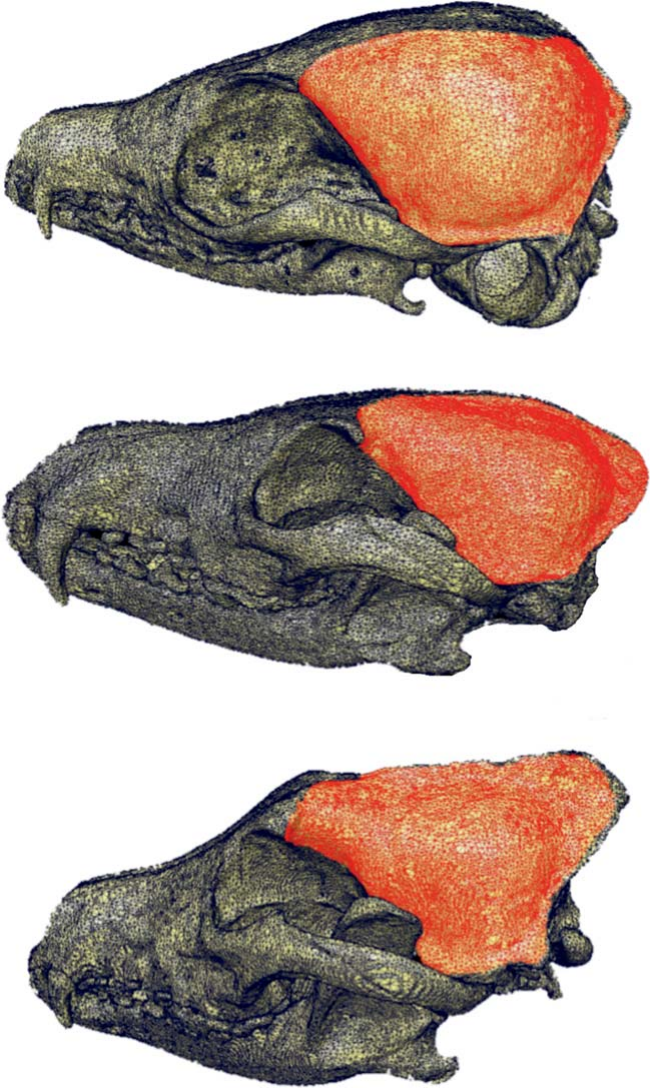
3D CT reconstructions demonstrating the area of origin of temporalis (red) on three species. **A**, *Vulpes zerda* exhibits a wide sagittal gap where left and right temporalis do not meet at midline, **B**, *Vulpes vulpes* demonstrates that the temporalis origin utilises all of the dorsal calvarium, and **C**, *Canis lupus*, displays a pronounced sagittal crest for increasing the surface area of temporal attachment.

**Figure 3:**
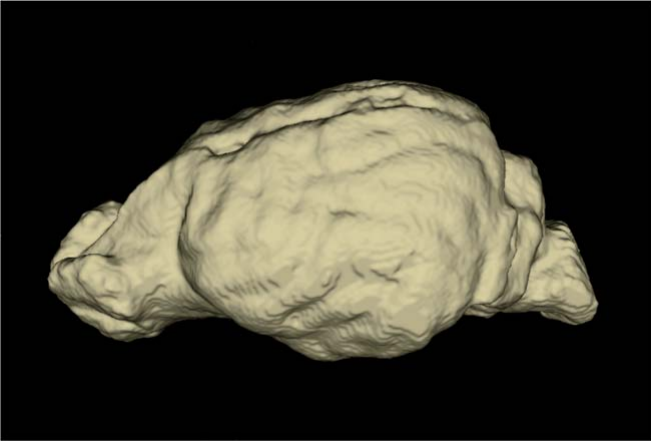
CT 3D reconstruction of *Chrysocyon brachyurus* endocast.

Surface areas for the muscle attachment sites were calculated in Avizo 8.1 by demarcating the bony boundaries of the muscle origins (Fig. 2). Endocranial volume (EV) is used as a proxy for brain volume and was calculated from CT images using the automatic segmentation 3D Active Contours function built into ITKsnap v2.4 (Yushkevich et al., 2006) (Fig. 3). In addition to endocranial volume, the endocranial volume surface area (EVSA) was calculated from the endocranial volume models using the Model module in 3D Slicer v4.3 (Fedorov et al., 2012). The EVSA values were then used as a proxy for the internal surface area of the cranium. This then allowed us to consider the internal surface area of the cranium and external attachment surface areas of the neurocranium as two separate variables.

### Statistics

Differences between the dietary groups regarding the percentage contribution of each muscle to the overall jaw adductor mass, and percentage contribution of each muscle division to the total muscle mass were tested using analysis of variation (ANOVA). To evaluate body size scaling trends log transformed values of muscle mass, muscle attachment surface area, endocranial volume, endocranial volume surface area and zygomatic arch width were regressed against body mass using the nonparametric Reduced Major Axes (RMA). RMA regression was used as there is measurement error in both variables. However, it is worth noting that whilst the non‐parametric RMA is the most appropriate method for these particular bivariate comparisons, the findings do not differ significantly from those calculated with ordinary least squares regression. Evaluations of isometry were made on the basis of the RMA slope 95% confidence intervals and *t* tests against predicted slope values. As temporalis is the largest of the jaw adductors, with the largest surface area attachment, we chose it to be the main focus of the accommodation part of this study.

Because the species in our samples may not be statistically independent due to a shared phylogeny, we repeated regressions with a phylogenetic independent contrast analyses based on an open access phylogenetic tree published by Nyakatura (Nyakatura and Bininda‐Emonds, 2012). The tree was pruned to include only our sample species (Appendix 2). Diagnostic tests were performed using the PDAP:Pdtree module v 1.16 in Mesquite v. 3.01 (Maddison and Maddison 2010) (Midford et al., 2005). Eleven variables representing species means were analysed (Table 2). We determined the absolute values of the standardized phylogenetic independent contrasts (PIC) for each character versus their standard deviations. A *P* value of ≤0.05 would be regarded as significant and would indicate phylogenetic influence (with values >0.05 indicating no phylogenetic signal). The reduced major axes regressions were also repeated using the PICs and the slopes compared against those for the standard data.

**Table 2:** Reduced major axes (RMA) regressions of variables scaled against body mass

		Standard RMA				PIC RMA	
Variable vs. log body mass (g)	Expected slope for isometry	RMA slope	95% conf. int. slope	*R* ^2^	*t* test	Slope	95% conf. int. slope
Log total adductor mass vs. Log BM	1	1.05	0.89,1.17	0.94	ns	1.03	0.92, 1.18
Log endocranial volume vs. Log BM	1	0.68	0.46, 0.77	0.90	***	0.60	0.47, 0.77
Log total surface area vs. Log BM	0.67	0.64	0.49,0.70	0.95	ns	0.59	0.50,0.67
Log temporalis SA vs. Log BM	0.67	0.64	0.49,0.70	0.95	ns	0.56	0.46, 0.67
Log masseter SA vs. Log BM	0.67	0.68	0.60, 0.72	0.98	ns	0.64	0.56, 0.70
Log pterygoids SA vs. Log BM	0.67	0.74	0.61, 0.82	0.93	ns	0.72	0.65, 0.83
Log temporalis mass vs. Log BM	1	1.05	0.87,1.16	0.93	ns	1.05	0.93,1.20
Log masseter mass vs. Log BM	1	1.1	0.94,1.20	0.95	ns	1.04	0.90,1.19
Log pterygoid mass vs. Log BM	1	0.97	0.84, 1.06	0.97	ns	0.89	0.77,1.05
Log zygomatic arch width vs. Log BM	0.33	0.32	0.27, 0.35	0.97	ns	0.31	0.26,0.36
Log EVSA vs. Log BM	0.67	0.43	0.31, 0.50	0.92	***	0.40	0.32, 0.51

a ns, not significant; *, *P* < 0.05; **, *P* < 0.01; ***, *P* < 0.001; SA, surface area; BM, body mass; EVSA, endocranial volume surface area.

Geometric morphometric analysis was used to identify and quantify patterns of morphological variation across species and between dietary niche groups. To ensure that all species had equal weighting in the analysis, one representative individual was chosen for each species. These individuals were identified from a preliminary morphometric analysis as the specimen closest to the mean shape for that species. The three‐dimensional coordinates for all sets of landmarks were imported into MorphoJ 1.45s and paired across the midline (Klingenberg, 2010). Generalized least squares full Procrustes fit was performed on all sets of data, which were then aligned by their principal axes. The asymmetric component of the shape change was briefly reviewed as it can highlight errors as well as asymmetries and the symmetric component was then further explored with a covariance matrix and principal components analyses to ascertain interspecific shape changes (Klingenberg, 2010). Scatterplots of the principal component (PC) scores were produced to visualize the distribution of datum points within the shape space, and wireframe models were created using key landmarks to visualize the range of shape deformation between the extremes. ANOVAs were used to test for significant differences between skull shapes (PC scores) and dietary groups. The pruned phylogenetic tree (Nyakatura and Bininda‐Emonds, 2012) was then mapped against the principal component datum points to indicate the overall influence of phylogeny on shape variation and permutation tests were performed on the null hypothesis of no phylogenetic signal (Klingenberg and Gidaszewski, 2010). Multivariate regression of the Procrustes coordinates against the body mass, where the shape landmark datasets were the dependent variables, tested for allometric signal, that is, the percentage of shape change that could be predicted by the change in body mass. Similarly, multivariate regression of the Procrustes coordinates of the shape landmark datasets on both temporalis mass and endocranial volume identified the percentage of shape change that was related to the change in temporalis mass and endocranial volume. The statistical significance of the regression analyses was tested with permutation tests against the null hypothesis of independence, and *P* values reported.

Reduced major axes regressions, analyses of variation (ANOVAs), post‐hoc Tukey analyses, and *t* tests were computed in PAST (Hammer et al., 2001). Mapping the phylogeny onto shape, and the multivariate regression of shape on body mass, temporalis mass and endocranial volume were computed in MorphoJ (Klingenberg, 2010). A significance level of 0.05 was used in all statistical tests.

## RESULTS

### Muscle Morphology

The masses (g) of the individual jaw adductor muscles and their subdivisions are presented in Table 1. Temporalis contributed between 57.4 and 69.3% to the total muscle mass (mean 62.1%), masseter contributed between 23.6 and 35.1% (mean 29.8%), and the pterygoids contributed between 6.7 and 10.5% (mean 8.2%). Next, individual muscles were considered. Temporalis was made up of three distinct divisions; suprazygomatic, superficial and deep temporalis. All species exhibited a well‐defined suprazygomatic portion of temporalis (Fig. 1b). This was consistently the smallest subdivision of temporalis, contributing 3.5–9.2% (mean 6.5%) of the overall temporal mass. Origin was by way of a short wide tendon arising from the temporal bone just dorsal to the external auditory meatus, and insertion was on the rostral aspect of the vertical ramus of the mandible. The remaining bulk of temporalis arises from the calvarium and divides into discrete superficial and deep parts. In the smallest species, *Vulpes zerda*, *Vulpes corsac*, and *Otocyon megalotis*, the origin of temporalis was lateral to midline. In all other species left and right temporalis met at midline, and in the larger species were associated with a pronounced sagittal crest (Fig. 2). Both the superficial and deep parts of temporalis insert onto the coronoid process and medial vertical ramus of the mandible (Fig. 1c,d). The superficial part of temporalis contributes between 39.7 and 59.5% (mean 46.5%) of the overall temporalis mass and the deep between 33.2 and 54.8% (mean 47.0%). The masseter is highly complex with more than the previously noted superficial, deep, and zygomaticomandibularis layers. The superficial division was well defined and contributes between 38.3 and 56.8% (mean 47.6%) to the overall masseteric mass. The origin is chiefly from the most ventral part of the zygomatic arch, but there is also a strong tendinous component originating dorsal to the upper molars (Fig. 1e,f). The caudal part of the superficial masseter extends beyond the caudal angle of the mandible to insert partly on the medial aspect of the mandible, and partly on the superficial aspect of the medial pterygoid (Fig. 1j). The deep masseter (Fig. 1g) originates from the medioventral aspect of the zygomatic arch. It is less clearly defined than the superficial division with many fibers arising or inserting onto aponeuroses within the muscle rather than directly to the bone of the mandible or zygomatic arch. It contributes between 12.2 and 36.4% (mean 24.1%) of the masseteric mass. Zygomaticomandibularis (Fig. 1h) originates from the caudal medial zygomatic arch and contributes between 16.7 and 44.0% (mean 28.3%) of the overall masseteric mass. Both of the pterygoid muscles were considered together as one muscle, the pterygoids, as the medial pterygoid was considerably more extensive than the lateral pterygoid (Fig. 1i). The (combined) pterygoids contributed between 7.0 and 10.5% of the total jaw adductor mass (mean 8.2%). The fascicles originate from the pterygoid plate of the skull and insert on the medial mandible. Temporalis arises from an extensive area of the lateral calvarium, in particular from the parietal, temporal, frontal and occipital bones (Fig. 2). The masseter arises from the ventral and medial borders of the zygomatic arch which itself is made up from the zygomatic and temporal bones, and the pterygoids arises from the sphenoid, pterygoid and palatine bones. Temporalis originates from a mean of 69.0% of the total jaw adductor attachment surface area, the masseter 18.6% and the pterygoids 12.0%.

### Metric Analysis

Results for the dietary group ANOVA tests revealed that there were no statistically significant differences between the dietary groups both for percentage contribution of each muscle to the overall jaw adductor mass, and percentage contribution of each muscle division to the total muscle mass (Table 3). Probability *P* values for the phylogenetic independent contrasts, comparing absolute values of the standardized phylogenetic independent contrasts versus their standard deviations ranged from 0.054 to 0.39. This suggests that phylogeny has a negligible effect. The RMA regressions on the pairs of variables that were generated with PICs showed no significant differences to those generated from the standard data, with similar slope and confidence intervals in all cases (Table 2). Phylogenetic influence on these variables and regressions is therefore considered minimal and subsequent allometric analyses focused on the raw metric data.

**Table 3:** ANOVAs for the differences between the dietary groups both for percentage contribution of each muscle to the overall jaw adductor mass, and percentage contribution of each muscle division to the total muscle mass

	*F* (2,9)	*P* value
Temporalis as a % of total jaw adductor mass	1.79	0.221
Masseter as a % of total jaw adductor mass	1.86	0.21
Pterygoids as a % of total jaw adductor mass	0.529	0.607
Suprazygomatic temporalis as % of total temporalis mass	0.089	0.924
Superficial temporalis as % of total temporalis mass	2.464	0.14
Deep temporalis as % of total temporalis mass	2.654	0.124
Superficial masseter as a % of total masseter mass	2.687	0.122
Deep masseter as a % of total masseter mass	2.208	0.166
Zygomaticomandibularis as a % of total masseter mass	2.408	0.145

**Figure 4:**
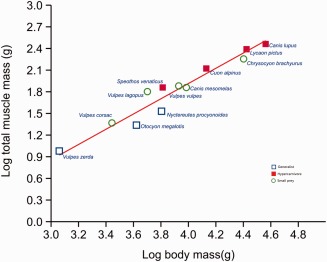
Reduced major axis regression, log body mass versus log total jaw adductor muscle mass. Dietary groups are highlighted.

**Figure 5:**
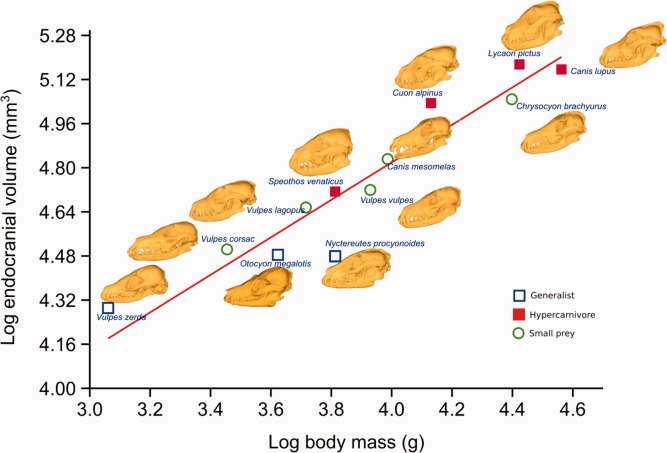
Reduced major axis regression, log body mass versus log endocranial volume. Dietary groups are highlighted and reconstructed skulls from CT scans illustrate variation in head shape.

Reduced major axis regressions of variables scaling against body mass are reported in Table 2. Scaling of total muscle mass was not significantly different to isometry and no trophic group appeared to deviate from this general scaling trend (Fig. 4). All three individual jaw adductor muscles scale close to isometry. Therefore species with a greater body mass have, in general, the same proportion of masticatory muscles to body mass as smaller species. Some regressions for muscle attachment surface area measurements may appear to be indicative of deviations from isometry (e.g., pterygoids and total muscle mass) but the confidence intervals encompass isometry in all cases and were not significantly different from isometry. Scaling of endocranial volume to body mass shows significant negative allometry (Fig. 5) with our results showing a slope of 0.68 from an expected isometric slope of 1 and a *t* test *P* value of 0.0007. That is, as species size increases the brain size increases to a lesser degree, and the brain takes up a lower proportion of overall body mass in large species of canids than in small ones. This was also reflected in the scaling of EVSA to body mass, which has a slope of 0.43 from an expected slope of 0.66 (*t* test *P* value 0.0001). The zygomatic arch width scales isometrically to body mass.

### Form Analyses

**Figure 6:**
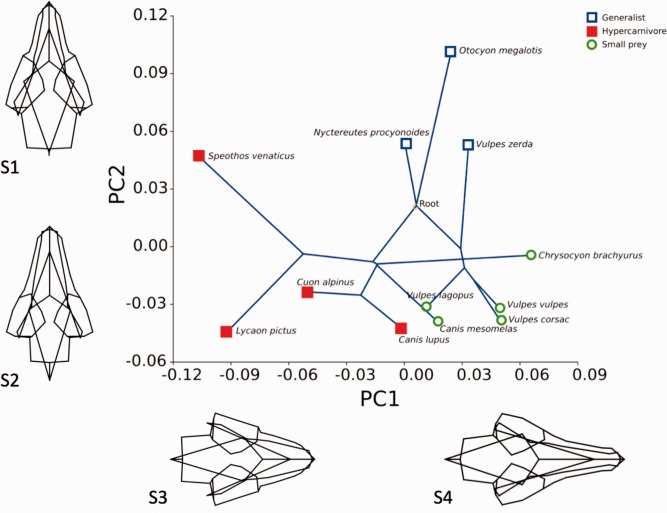
Whole skull principal component scores PC1 versus PC2. Dietary groups and the mapped phylogenetic tree is shown within the plot and wireframes representing skull shape changes are aligned along the relevant axes.

In the whole skull landmark data set, the first 4 PCs make up 78.5% of the variance. PCs 1 and 2 are described in detail and shown in Fig. 6. PC1 constituted 32.3% of shape variance. At the negative extreme of the axis (−0.11, represented by wireframe S3) the rostral landmarks move caudolaterally, resulting in an overall shape change of a shorter broader snout and mandible. The landmarks associated with the zygomatic arches and caudal mandible move laterally, representing a relative broadening of the skull, and the dorsal landmarks of the inion and dorsal skull move dorsally—a shape change associated with a larger sagittal crest. Landmarks relating to the ventral aspect of the skull move ventrally resulting in an overall deepening of the cranium. At the other extent of the axis (0.07, represented by wireframe S4), the snout and mandible become longer and more gracile and the cranium appears dorsoventrally flattened. The PC1 axis clearly differentiated the data into dietary groups. The species occupying the lower end of the range (−0.12 to −0.01) were exclusively hypercarnivorous, the generalists occupied the middle zone and (0 to 0.03) and the small prey specialists the higher end of the range (0.015–0.07) with some overlap of the generalist species at their lower values. The ANOVA for the whole skull PC1 scores shows significant difference between the dietary groups, *F* = (2,9) = 12.29; *P* = 0.003. Tukey's pairwise post hoc tests showed that the hypercarnivores were significantly different to both small prey (*P* value 0.004) and generalists (*P* value 0.013). Of particular note are *Speothos venaticus*, a small hypercarnivorous canid that lies with the other three hypercarnivores at the low value extreme of this axis despite weighing only 6.5 kg, and its close relative, *Chrysocyon brachyurus*, a 22.5‐kg specimen, that lies with the *Vulpes* group at the other extreme of the axis. PC2 makes up 25.5% of variance. At one extreme (−0.06, represented by wireframe S2) the cranium appears relatively shorter and more domed and the dorsal border of the mandible is straighter. At the other extreme, (0.11, represented by wireframe S1) the cranial component appears dorsally flattened and elongated and the dorsal border of the mandible is curved. The ANOVA for the whole skull PC2 scores shows significant difference between the dietary groups, *F* = (2,9) = 11.24; *P* = 0.004. Tukey's pairwise post hoc tests showed that the hypercarnivores were significantly different to the generalists (*P* value 0.008) and that the generalists were significantly different to the small prey specialists *(P* value 0.003).

**Figure 7:**
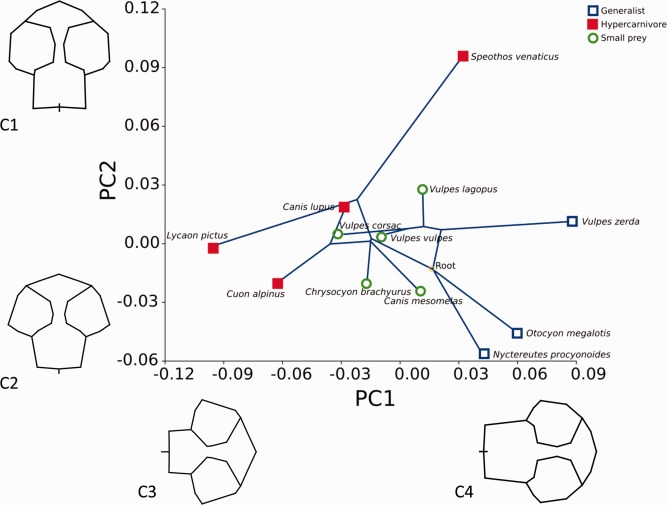
Cranial principal component scores PC1 versus PC2. Dietary groups and the mapped phylogenetic tree is shown within the plot and wireframes representing skull shape changes are aligned along the relevant axes.

In the cranial subset (32 landmarks) the first 4 PCs make up 76.8% of the variation: PCs 1 and 2 are described in detail and shown in Fig. 7. PC1 constituted 33.1% of the shape variance. At the negative extreme of the axis (−0. 11, represented by wireframe C3), caudal landmarks move rostrally and dorsal landmarks move dorsally resulting in a relatively shorter deeper skull. Lateral landmarks moving laterally achieve relative widening of the zygomatic arch. At the positive extreme of the axis (0.09, represented by wireframe C4), the cranium lengthened whilst the zygomatic arches became relatively narrower. All dorsal landmarks shifted ventrally, resulting in a flatter skull. The relatively ventral position of the inion indicates a small or absent sagittal crest. The PC1 axis showed some differentiation of the data into dietary groups. At the negative end of the axis were 3 of the hypercarnivores and at the other, the generalists. The small prey specialists occupied the middle space with some overlap with the hypercarnivores. *Speothos venaticus*, the fourth hypercarnivore appeared between the small prey specialists and generalists. The ANOVA for the cranial PC1 scores shows significant difference between the dietary groups *F* = (2,9) = 7.38; *P* = 0.01). Tukey's pairwise post hoc tests showed that the hypercarnivores were significantly different to the generalists (*P* value 0.008). PC2 made up 19.6% of the shape variance. At the negative extreme of the axis (−0.06, represented by wireframe C2), the zygomatic landmarks move dorsally and the dorsal landmarks move ventrally. At the positive end of the axis (0.11 represented by wireframe C1) the zygomatic landmarks move ventrally and the dorsal landmarks move dorsally. However, only one specimen, *Speothos venaticus*, lay towards the extreme end of the positive axis, all other specimens were closely grouped between −0.06 and 0.03. PC2 showed no appreciable grouping of species into dietary specialisms and ANOVA tests showed no significant differences between the dietary groups.

**Figure 8:**
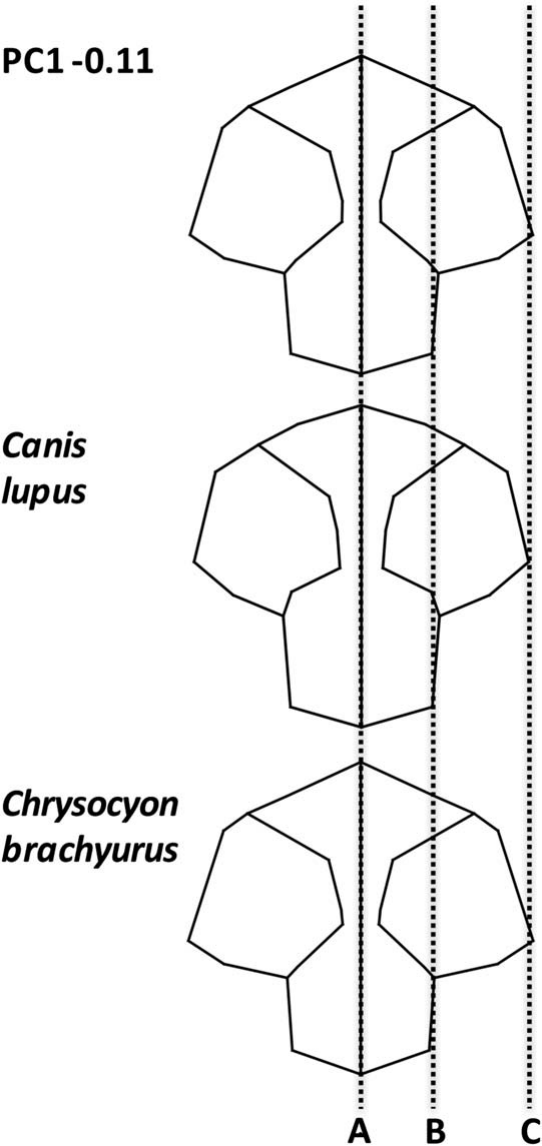
Dorsoventral view of cranial wireframes comparing the −0.11 principal component analysis with that of *Canis lupus* and *Chrysocyon brachyurus*. Line “A” represents the midline, and line “C” the lateral extent of the zygomatic arch. Line “B” represents the lateral extent of the cranium.

When the pruned phylogenetic tree was mapped onto the PC scores it showed that the whole skull, rostral and mandibular component analyses contained phylogenetic signal, with *P* values from 0.009 to 0.014, whereas the cranial subset demonstrated no statistically significant phylogenetic signal (Table 4). This indicates that the rostral and mandibular components of the skull are strongly linked to phylogeny, whereas the form of the cranial component changes in response to other constraints. The overall phylogenetic maps (Figs. 6 and 7) show some long terminal branches, compared to shorter internal branches, indicating that some closely related species have diverged considerably within the shape space demonstrating substantial differences in related morphologies. In tests for allometric signal, regression of the cranial component shape on body mass showed the greatest percentage (16.5%) of shape variance of any of the landmark sets, and was the only landmark dataset with a statistically significant permutation *P* value (Table 4). The cranial component thus demonstrates evidence against the null hypothesis of complete independence, suggesting that shape change is related to size change. The multivariate regression analyses of shape on temporalis mass shows that the highest percentage change relates to the cranial shape dataset and was statistically significant. Similarly the highest percentage shape change linked to endocranial volume was also the cranial dataset, and was also statistically significant. This demonstrates that change in temporalis mass and endocranial volume are linked with change to cranial shape (Table 4). Figure 8 compares the cranial wireframe shapes of two distantly related species with the cranial wireframe shape representative of the low PC1 score. The low PC1score wireframe indicates a short deep skull with increased space medial to the zygomatic arches for housing the temporalis muscles. Although *Canis lupus* and *Chrysocyon brachyurus* are from different clades and exhibit distinct dietary preferences and hunting strategies both have large body masses and relatively small endocranial volumes. The wireframes indicate that in both species cranial shape is very similar, both to each other and to the PC1 wireframe. The remaining three shape datasets shapes showed lower percentage changes and had no statistical significance, indicating that overall head shape, rostral shape and mandibular shape changes are independent of temporalis mass or endocranial volume change.

**Table 4:** Summary statistics for form analyses

	Phylogenetic signal	Allometric signal		Effect of temporalis mass on cranial shape		Effect of endocranial volume on cranial shape	
Form	*P* value (<0.05 indicates phylogenetic signal)	% Shape change	Permutation test *P* value	% Shape change	Permutation test *P* value	% Shape change	Permutation test *P* value
Whole skull	**0.009**	11.0	0.26	14.7	0.11	16.3	0.053
Cranial component	0.053	16.5	**0.04**	19.1	**0.01**	22.4	**0.002**
Rostral component	**0.012**	11.9	0.24	14.6	0.15	16.2	0.11
Mandibular component	**0.014**	9.1	0.39	12.9	0.19	15.1	0.118

Significant values are shown in bold.

## DISCUSSION

Our results show that the morphology of the jaw adductor muscles is remarkably conserved across canid species. The form of each muscle and its subdivisions were surprisingly similar in all cases given the diverse dietary niches, different body sizes and phylogeny. We also found that the jaw adductor mass as a whole, and all three of the jaw adductor muscles individually, scale isometrically. Although we reported a couple of differences of muscle subdivision scaling between the different dietary groups, non were statistically significant and morphological variance was minimal and much less than we expected—that is, hypercarnivorous species, which might be expected to have a have relatively larger muscles to generate greater bite force, have the same ratio of muscle masses to body mass as those with assumed weaker bites, the generalists and small prey hunters. All individuals were consistent with the scaling pattern and there were no correlations relating to phylogeny or dietary groups (Fig. 4). Skull shape variation is therefore not attributable to housing differently scaled muscle masses for specialist dietary or different phylogenetic groupings. Whilst our sample sizes per species were relatively modest and were not sex matched, there were large scale interspecific differences and evidence from previous studies (see Methods) suggests that there is minimal sexual dimorphism.

**Figure 9:**
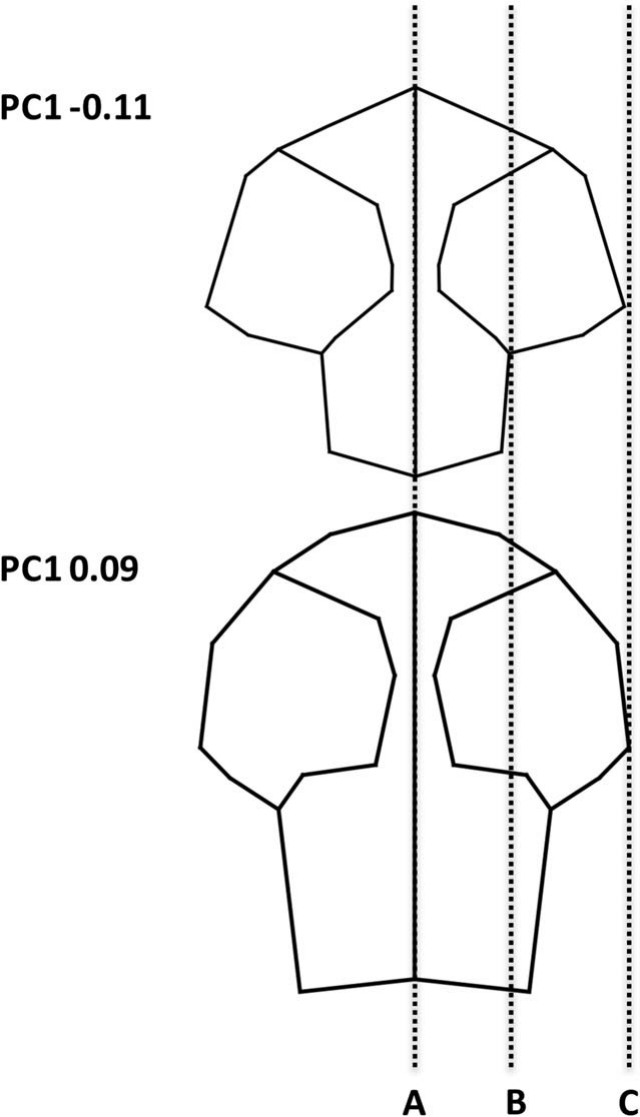
Dorsoventral view wireframes taken PC1 of the cranial component analysis. Both diagrams have been scaled to have equal zygomatic width as this scales isometrically. Line “**A**” represents the midline and line “**C**” the lateral extent of the zygomatic arch. Line “**B**” represents the lateral extent of the cranium in the species with low PC1 scores.

Our endocranial volume scaling results are in accordance with previous studies (Jerison, 1955; Gould, 1966; Bauchot, 1978) that describe interspecific scaling at a rate of two thirds relative to body mass. This presents the problem of accommodating isometrically scaling muscle masses onto negatively scaling neurocrania. We considered the cranium to have two discrete surface areas: an internal one which reflects the accommodation needs of the brain and which was calculated as the surface area of the endocast (EVSA), and an external one, which was calculated as the area of origin for temporalis (we acknowledged that this only accounts for part of the external surface of the cranium). The EVSA scales to body mass with marked negative allometry, whilst the scaling of temporalis surface area to body mass is not significantly different to isometry. The disparity between the demands of the internal and external surfaces of the neurocrania was further evidenced by the very small canids displaying a sagittal gap at dorsal midline where there is no muscle attachment, demonstrating that in these species the external surface of the cranium more closely reflects its internal surface area which is driven by brain accommodation. The sagittal gap was only seen in species below 5kg, that is, *Vulpes zerda*, *Vulpes corsac*, and *Otocyon megalotis*. From 5 to 10 kg the contralateral temporalis muscles met at midline as temporalis utilised all of the available external surface area, with little or no sagittal crest present. Above 10 kg a pronounced sagittal crest was seen which increased the surface area available for temporalis (Fig. 2). The exception to this was the 6.5 kg *Nyctereutes procyonoides* that also had a well‐developed sagittal crest. However, this was also the species with the smallest endocranial volume relative to body mass, as evidenced by the greatest negative distance from the regression line for endocranial volume against body mass (Fig. 5). By contrast, the *Nyctereutes* temporalis surface area scales at a similar rate to other species and so the sagittal crest demonstrably increases the external surface area commensurate with temporalis requirements. These findings suggest that the exterior surface area of the calvarium does not simply reflect the interior, but is driven by the necessity to accommodate the temporalis and probably the other muscles too. The other major morphological adaptation to increase the space on the skull for housing the temporalis is the isometrically scaling zygomatic arches (Fig. 9). Other authors (Radinsky, 1981; Emerson and Bramble, 1993) have speculated that if the arch width remains relatively constant but the endocranial volume decreases relatively, the space medial to the arches increases and could be used to accommodate a larger temporalis. Our studies have shown that this “increased accommodation” principle is correct but that in canids, the space is utilized to house an isometrically, rather than positively, scaling temporalis. Interestingly, primates have also been shown to have isometrically scaling masticatory muscles (Cachel, 1984) as well as negatively scaling endocrania (Rilling, 2006) and large species of primate exhibit similar morphological features as large species of canid, such as sagittal crests (Ankel‐Simons, 2007) and relatively wide zygomatic arches (Frost et al., 2003). This might suggest that the problem of muscle accommodation is more universal than indicated here, although it is important not to extrapolate our findings too far as the two groups have, for instance, distinct dietary behaviors.

Principal component analysis of the whole skull form differentiated the species into the three broad dietary groups and in the multivariate regression analyses demonstrated phylogenetic signal but no allometric signal. Total skull shape aligned broad stocky head shapes with hypercarnivourous hunters, and narrow slender head shapes with the small prey specialists. The generalists lay in the middle ground. This is in agreement with previous studies (Wroe and Milne, 2007; Goswami et al., 2011). However, when we removed all of the rostral and mandibular components and focused only on the cranial component, there was no significant phylogenetic signal, dietary specialism grouping was less marked and a significant allometric signal indicated that shape change was related to size change. More specifically, cranial shape changes correlate with body mass, temporalis mass and endocranial volume changes. Shorter, dorsoventrally enlarged calvaria, increased sagittal crests and widened temporal spaces correspond with the decreasing endocranial volume to temporalis ratio that is seen as body mass increases. This directly links shape change in the cranium with accommodation of temporalis, and suggests that the rostral and mandibular components are chiefly concerned with dietary specialism, whilst the cranial component is more strongly associated with muscle accommodation. In the whole skull analysis (Fig. 6), *Chrysocyon brachyurus*, for example, is closely aligned with the *Vulpes* group at the furthest distance from the hypercarnivore species. All species at the positive end of the axis exhibit the long narrow jaws of the small prey hunter, and in the case of *Chrysocyon brachyurus*, the crossing tree branches also demonstrate convergent evolution (Gidaszewski et al., 2009). However, when we focus on the cranial component (Fig. 7) *Chrysocyon* shape is more closely aligned with the hypercarnivores, due to the large sagittal crest and wide medial zygomatic space that accommodates the temporalis. It is presumed that some elements of cranial shape change will not directly associate themselves with muscle accommodation, but may be linked with considerations other than scaling. Such factors may include generating biomechanical advantage to facilitate certain bite behaviors such as fast jaw snapping, or increasing bite force. Similarly, other biomechanical functions of the skull such as withstanding stress or dissipating bite forces have not been considered in this study. These factors warrant further consideration in order to understand how canids have applied similar muscle proportions in the generation of different bite forces and speeds, to occupy remarkably distinct dietary niches.

### Summary

There are two main factors that influence shape change in the canid skull: features that are scaled relative to body mass (whether isometrically or allometrically), and features that change independently of body mass. Our findings show that the jaw adductor muscles scale isometrically to body mass, even though they are functionally aligned to independently changing features such as jaw length or bite force, both of which are allied to dietary specialisms (Christiansen and Wroe, 2007; Van Valkenburgh, 2007; Figueirido et al., 2011; Damasceno et al., 2013). Our results suggest that much of the cranial shape change is related to accommodating temporalis. These findings may help inform work on interpreting the feeding habits of extinct species (e.g., Wroe et al., 2005; Meloro et al., 2015). It should be noted, however, that our findings do not preclude the cranial shape changes also being biomechanically advantageous to the different trophic groups. In future work we hope to consider how the architectural details of the jaw adductor muscles such as fascicle orientation, fascicle length and angles of pennation, may affect muscle force capability, and how the spatial relationships between muscle centroid size and key skull features such as the temporomandibular joint and carnassial or canine teeth, influence bite force.

## APPENDIX 1 Geometric morphometric landmarks

**Table:**

Cranial component	Rostral component	Mandibular component
Caudal zygomatic temporal junction	Rostral alveolus of upper canine	Rostral alveolus of lower canine
Rostral zygomatic temporal junction	Caudal alveolus of upper canine	Caudal alveolus of lower canine
Lower rostral zygomatic ridge	Rostral alveolus of upper carnassial	Rostral alveolus of lower carnassial
Lateral articular mandibular condyle	Caudal alveolus of upper carnassial	Caudal alveolus of lower carnassial
Medial articular mandibular condyle	Caudal upper tooth row	Caudal lower tooth row
Ventral retroglenoid process	Lateral point of infraorbital foramen	Lateral point of rostral mental foramen
Pterygoid process	Zygomatic maxillary junction	Rostroventral masseteric fossa on mandible
Tympano‐occipital fissure, medial point	**Inter‐incisive**	Angular process on mandible
Rostral pterygoid ridge	**Rostral internasal**	Caudal coronoid process
Caudal pterygoid ridge	**Nasion**	Medial point of inferior alveolar foramen
Mid pterygoid ridge	**Caudal hard palate**	**Rostral mandibular symphysis**
Zygomatic process of frontal bone		**Caudal mandibular symphysis**
Caudal ventral jugular process		
Cochlear apex		
**Nasion**		
**Basion**		
**Dorsal foramen magnum**		
**Inion**		

^1^Bold type indicates unpaired (sagittal) landmarks.
